# 2-Aminoacrylate stress damages diverse PLP-dependent enzymes *in vivo*

**DOI:** 10.1016/j.jbc.2022.101970

**Published:** 2022-04-20

**Authors:** Wangchen Shen, Andrew J. Borchert, Diana M. Downs

**Affiliations:** Department of Microbiology, University of Georgia, Athens, Georgia, USA

**Keywords:** 2-aminoacrylate damage, pyridoxal 5′-phosphate enzymes, metabolite stress, 2AA, 2-aminoacrylate, PLP, pyridoxal 5′-phosphate

## Abstract

Pyridoxal 5′-phosphate (PLP) is an essential cofactor for a class of enzymes that catalyze diverse reactions in central metabolism. The catalytic mechanism of some PLP-dependent enzymes involves the generation of reactive enamine intermediates like 2-aminoacrylate (2AA). 2AA can covalently modify PLP in the active site of some PLP-dependent enzymes and subsequently inactivate the enzyme through the formation of a PLP–pyruvate adduct. In the absence of the enamine/imine deaminase RidA, *Salmonella enterica* experiences 2AA-mediated metabolic stress. Surprisingly, PLP-dependent enzymes that generate endogenous 2AA appear to be immune to its attack, while other PLP-dependent enzymes accumulate damage in the presence of 2AA stress; however, structural determinants of 2AA sensitivity are unclear. In this study, we refined a molecular method to query proteins from diverse systems for their sensitivity to 2AA *in vivo*. This method was then used to examine active site residues of Alr, a 2AA-sensitive PLP-dependent enzyme, that affect its sensitivity to 2AA *in vivo*. Unexpectedly, our data also showed that a low level of 2AA stress can persist even in the presence of a functional RidA. In summary, this study expands our understanding of 2AA metabolism and takes an initial step toward characterizing the structural determinants influencing enzyme susceptibility to damage by free 2AA.

The metabolic network in microbes is responsible for the robust physiology characteristic of these organisms. A responsive metabolism can react to perturbations caused by both internal and external stresses and maintain overall fitness of the organism. Included in the metabolic network are numerous enzymes that utilize a range of cofactors to catalyze the biochemical reactions necessary for an organism to thrive. Disruption of these reactions causes perturbations throughout the system and can result in detectable phenotypic consequences. One means by which enzymatic reactions can be compromised is to limit or damage a required cofactor. Relevant to the work herein is pyridoxal 5′-phosphate (PLP), an essential cofactor for a class of enzymes most often involved in transamination, decarboxylation, and racemization reactions (1). Discovery and characterization of the RidA enamine/imine deaminase defined a metabolic stress that is generated by the reactive intermediate, 2-aminoacrylate (2AA). Significantly, 2AA was both generated by, and caused damage to, PLP-dependent enzymes (reviewed in the studies by Borchert *et al.* (2) and Irons *et al.* (3)).

The catalytic mechanism of some PLP-dependent enzymes (*i.e.*, threonine/serine dehydratases [EC 4.3.1.19]) generates a reactive enamine intermediate that is protonated to an iminium ion, which then undergoes Schiff base hydrolysis to form a final relevant product. For instance, the biosynthetic serine/threonine dehydratase, IlvA (EC 4.3.1.19), dehydrates L-threonine to the enamine intermediate 2-aminocrotonate, which is hydrolyzed to 2-ketobutyrate (releasing ammonium) as the first step in L-isoleucine biosynthesis (4, 5). Previous work investigating the stereospecific deuteration of 2-ketobutyrate formed by IlvA from *Serratia marcescens* found that protonation of 2-aminocrotonate was enzyme catalyzed (6). However, *Salmonella enterica* and *Escherichia coli* IlvA orthologs generate 2-aminocrotonate that can interact with phosphoribosyl pyrophosphate in an anthranilate phosphoribosyltransferase–dependent manner to form phosphoribosyl amine, an intermediate in purine biosynthesis (7, 8). These findings suggest that IlvA orthologs from *S. enterica* and *E. coli* either do not catalyze the protonation of 2-aminocrotonate or, if 2-aminocrotonate protonation is enzyme catalyzed, the imine product can be released from the active cite and undergo tautomerization back to 2-aminocrotonate. IlvA can also use L-serine as a substrate, which generates 2AA, a reactive enamine with a half-life of less than 3 min in aqueous solution (9, 10). Significantly, 2AA damages some PLP-dependent enzymes by a covalent modification which involves forming a pyruvate–PLP adduct and thus inactivating the enzyme (11, 12) (Fig. 1).

**Figure 1:**
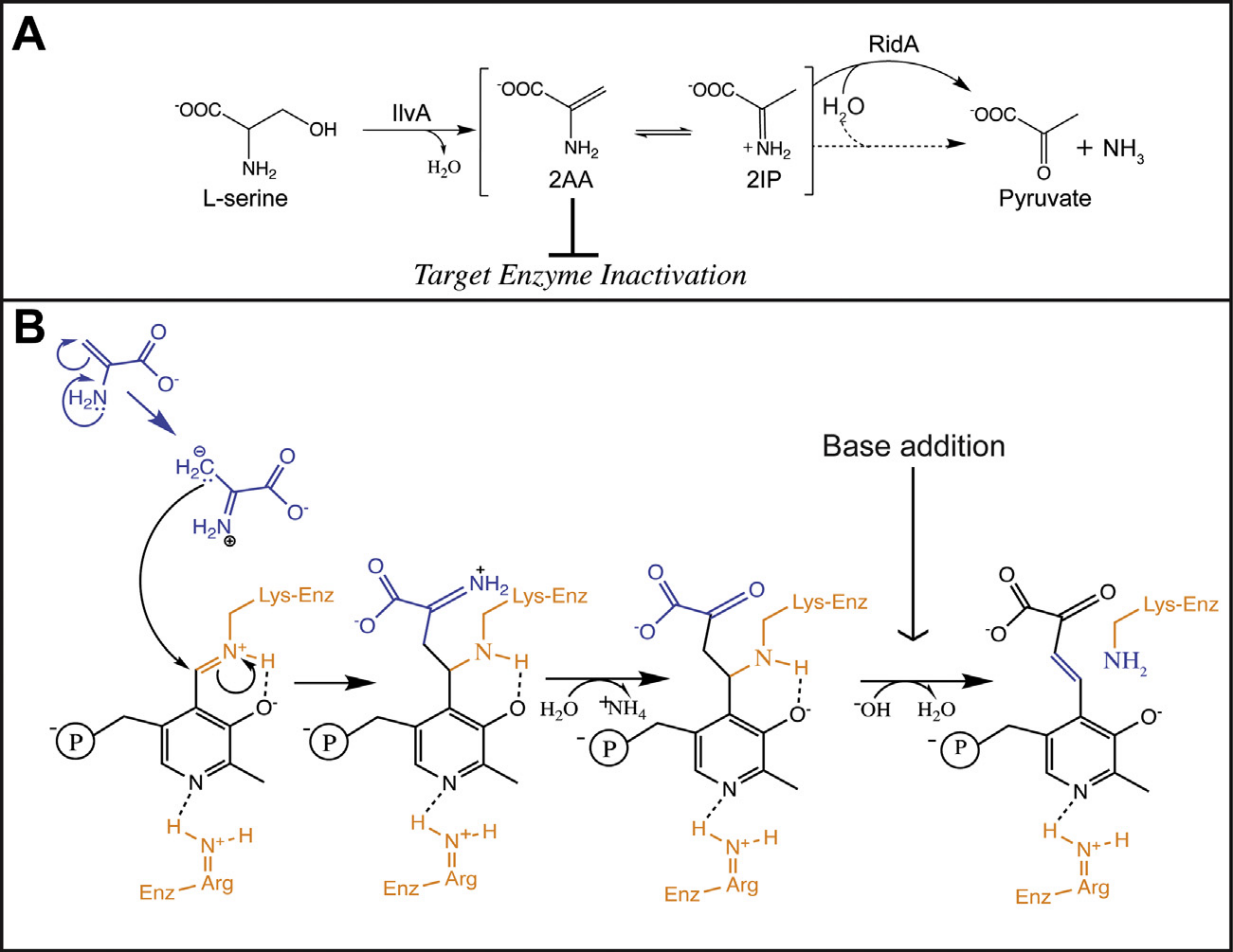
**Endogenous 2AA formation and attack.***A*, reaction mechanism of serine/threonine dehydratase (IlvA) using serine as a substrate. *B*, pyruvate–PLP adduct is formed by 2-aminoacrylate attacking an active site PLP. Shown in the mechanism by which pyruvate–PLP is formed. A fold type III active site is represented with arginine as the residue coordinating the pyridine nitrogen of PLP (ala alanine racemase). The mechanism of attack has been described (2). Treatment with base releases a pyruvate–PLP adduct that can be easily separated from PLP and monitored by HPLC. 2AA, 2-aminoacrylate; 2IP, 2-iminopropanoate; PLP, pyridoxal 5′-phosphate.

RidA is a broadly conserved member of the large Rid (YjgF/YER057c/UK114) protein superfamily (13). Members of the RidA subfamily deaminate multiple enamines/imines to the corresponding ketoacids with a faster rate than can be mediated by solution water (14, 15). Characterization of a *ridA* mutant of *S. enterica* showed that despite its short half-life, 2AA could persist *in vivo* and partially inactivate certain cellular PLP-dependent enzymes (14, 16, 17). The metabolic defects associated with 2AA persistence in a *ridA* mutant are exacerbated by L-serine, due to increased formation of 2AA by IlvA (18, 19, 20, 21).

Most PLP-dependent enzymes can be grouped into four distinct fold types based on their structural similarities and evolutionary relatedness (1, 22). The dominant 2AA generator in *S. enterica*, IlvA, is fold type II, as are two other dehydratases that generate 2AA in this organism, cysteine desulfhydrase CdsH (EC 2.5.1.47) and diaminopropionate ammonia-lyase DapL (EC 4.3.1.15) (23, 24). The finding that these enzymes generate 2AA that can diffuse out of the active site suggested that they were at least partially immune to attack by this enamine. Covalent inactivation of PLP-dependent enzymes by 2AA has been characterized *in vitro* (11, 12) and *in vivo* when RidA is absent (14, 25, 26). *In vivo* targets of 2AA have been identified in bacteria and yeast and include serine hydroxymethyl transferase (EC 2.1.2.1) (27) (fold type I), alanine racemase Alr (EC 5.1.1.1) (fold type III) (16), branched-chain amino acid aminotransferase (EC 2.6.1.42) (fold type IV) (14, 21), aspartate transaminase (EC 2.6.1.1) (fold type I) (20), and aminolevulinic synthase (EC 2.3.1.37) (fold type II) (26, 28, 29). Many more targets of 2AA stress are expected given the numerous PLP-dependent enzymes in most organisms.

In the studies of RidA, a rough correlation between fold type and the ability of PLP-dependent enzymes to generate, or be inactivated by, 2AA seemed to exist. Namely, enzymes belonging to the fold type II class of PLP-dependent enzymes appeared resistant to 2AA damage, while those were sensitive to damage belonged to other fold types. The present study was initiated to implement a system to detect and quantify damage caused by 2AA *in vivo*. The work herein explored structural features of an enzyme that affect sensitivity to 2AA and developed a system to quantify *in vivo* 2AA stress that might not result in detectable phenotypic consequence(s).

## Results

### A pyruvate–PLP adduct forms in Alr from *ridA* cells

A previous study found a fraction of alanine racemase, Alr, purified from a *ridA* mutant carried a pyruvate–PLP adduct, indicating prior attack by 2AA. In contrast, a pyruvate–PLP adduct was absent from the same protein purified from an isogenic wildtype strain (16). Distinct strains and slightly modified protocols were used here to affirm this result and extend the understanding of the 2AA stress present *in vivo*. An isogenic pair of strains carrying either a wildtype (DM13509) or null allele of *ridA* (DM17050) was used as host for a set of plasmids expressing Alr^WT^ or select Alr variants. Strains were grown in minimal medium supplemented with L-serine (5 mM) and glycine (1 mM); these additions ensured 2AA production and full growth in the presence of 2AA stress, respectively (17, 18). The respective Alr protein was overexpressed and purified from both the wildtype and ridA mutant strains. Each protein sample was treated with base, and the released cofactors were separated by high-performance liquid chromatography (HPLC) and monitored at 305 nm (16).

**Figure 2:**
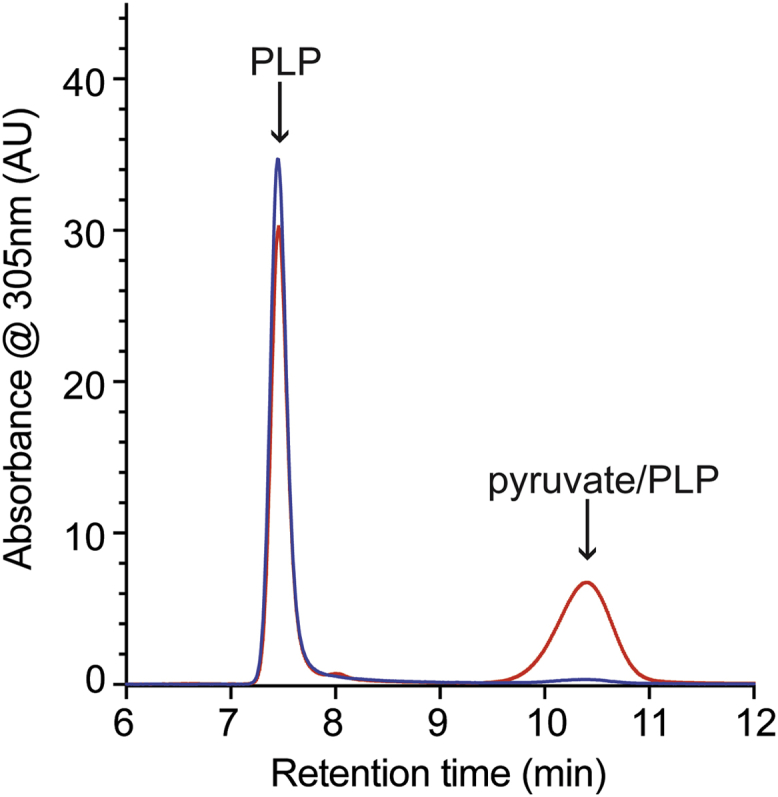
**Alr is damaged when 2AA is present *in vivo*.** Wildtype Alr was purified from wildtype (*blue*) and a *ridA* mutant (*red*) of *S. enterica* as described in the text. Cofactors from 0.4 mg of enzyme were released by treatment with base and separated by HPLC. Cofactors are labeled based on retention time and coinjection experiments with authentic standards. 2AA, 2-aminoacrylate.

The HPLC chromatogram derived from the Alr^WT^ protein sample purified from the *ridA* mutant strain had two dominant peaks (Fig. 2). The first peak had a retention time close to 8 min and could be attributed to PLP (Fig. S1). The second peak had a retention time of ∼10.5 min and was ascribed to a pyruvate–PLP adduct (Fig. S1). The sample derived from the wildtype strain had a large PLP peak at ∼8 min and little, if any, detectable absorbance (≤500 AU) at 305 nm between 10 and 12 min. The released cofactors were quantified using peak area measured at the relevant absorbance maximum, and a standard curve was generated with the authentic metabolites (Fig. S2). Analyses of the peak areas indicated 0.83 ± 0.01 and 0.66 ± 0.03 nmol PLP were released from 1 nmol of Alr protein purified from the wildtype or *ridA* mutant, respectively. Approximately 0.11 ± 0.01 nmol pyruvate–PLP was released per nmol Alr protein when purified from the *ridA* mutant, while pyruvate–PLP adduct was undetectable when treating Alr purified from the wildtype strain. The pyruvate–PLP accounted for approximately 14% of the total cofactor released from the Alr^WT^ protein isolated from the *ridA* mutant strain. The specific activity of Alr protein samples purified from wildtype and the *ridA* mutant was 2.52 ± 0.05 and 2.17 ± 0.22 μmol alanine min^−1^ mg^−1^, respectively. Taken together, these data suggested the 2AA-dependent modification was responsible for the decreased activity of Alr in a strain lacking RidA, as previously proposed (16). Further, these data suggested a framework to probe the direct impact of *in vivo* 2AA stress on PLP-dependent proteins by assessing pyruvate–PLP released from a protein sample.

### A pyruvate–PLP adduct accumulates in multiple Alr variants

**Figure 3:**
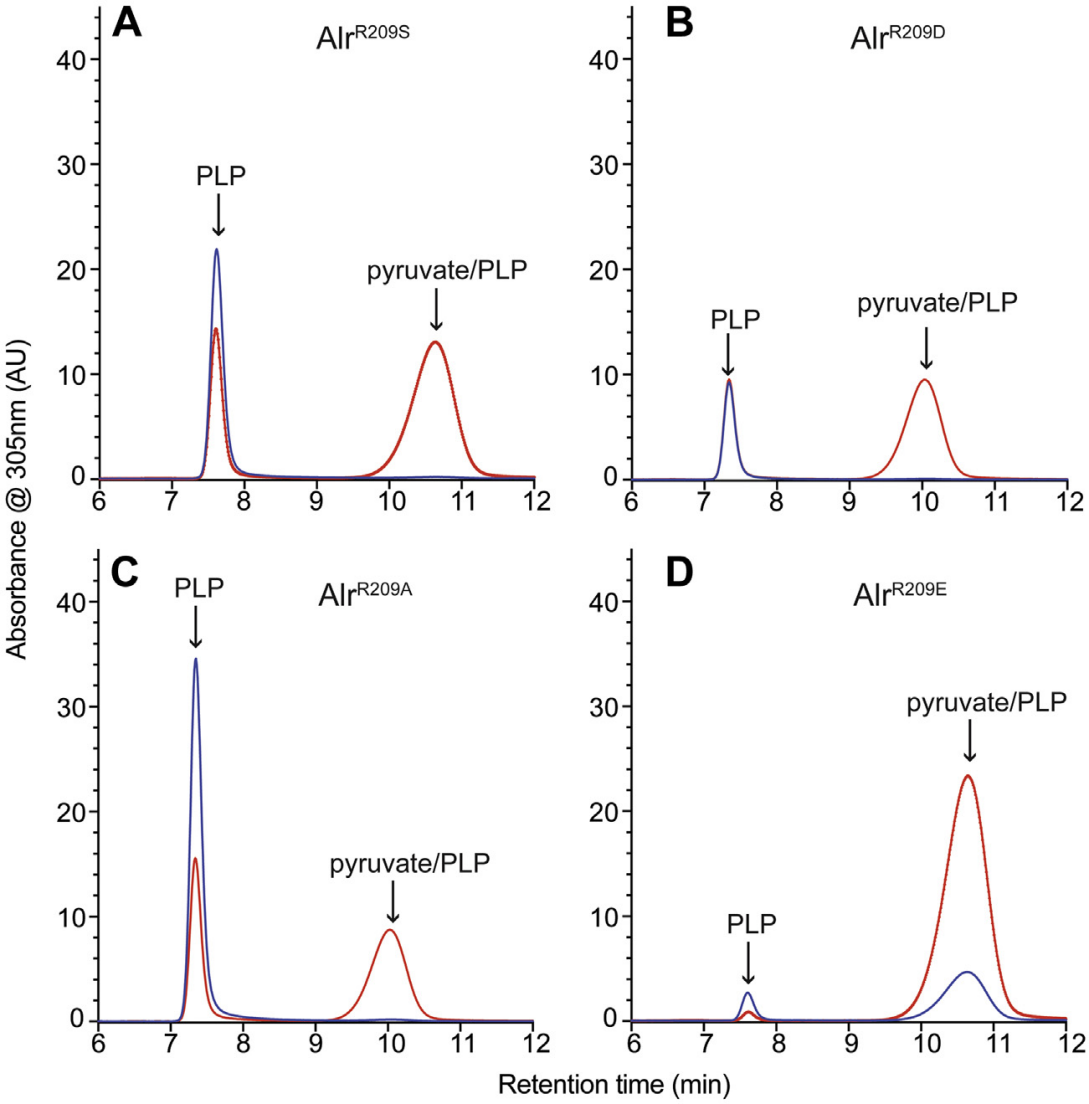
**Alr variants have increased damage from 2AA.** Indicated protein variants (*A-D*) were purified from a wildtype strain (*blue*) or a *ridA* mutant (*red*) of *S. enterica*. Cofactors from 0.4 mg of enzyme were released with base treatment and separated by HPLC. Cofactors are labeled based on retention time and coinjection experiments with authentic standards. 2AA, 2-aminoacrylate.

To probe the susceptibility of Alr variants to 2AA damage, we focused on the residue coordinating the pyridine nitrogen of the bound PLP. This residue has been implicated in assignment of fold type and appears to exert significant control over differences in the electrophilic strength of the Schiff base in PLP-dependent enzymes (30, 31). In a simple scenario, such a residue (R209 in Alr) could affect the interaction of 2AA with the active site PLP to impact susceptibility to attack. Based on this reasoning, Alr variants with substitutions at R209, designed to imitate different fold types and change the electrophilicity of the Schiff base, were constructed. Fold type II includes proteins shown to generate 2AA stress *in vivo* and have a serine or threonine residue in the position equivalent to 209. An Alr^R209S^ variant was generated and queried for 2AA damage in the relevant genetic backgrounds (Fig. 3*A*). Similar to Alr^WT^, the Alr^R209S^ variant purified from a wildtype strain released PLP and no detectable pyruvate–PLP. However, the Alr^R209S^ variant purified from the *ridA* mutant background released considerably more pyruvate–PLP than the wildtype protein (0.55 *versus* 0.11 nmol/nmol protein, respectively), and perhaps more significantly, the pyruvate–PLP made up 66% of the total cofactor released from this variant. Both samples of the Alr^R209S^ variant released less PLP than the wildtype protein, most likely a consequence of the changed active site environment. These data suggest the Alr^R209S^ variant is more susceptible to 2AA damage than the wildtype protein *in vivo*, despite having a substitution making it more like fold type II enzyme.

Additional Alr variants substituted R209 with other residues to alter the electrophobicity of the N Schiff base (32). Alr^R209D^, Alr^R209E^, and Alr^R209A^ variants were similarly purified from a strain in which 2AA persisted (ridA) and one where it would not (wildtype). All three variants had evidence of attack by 2AA *in vivo* reflected in the release of pyruvate–PLP from protein purified from a *ridA* mutant (Fig. 3 and Table S1). Particularly telling was the high percentage of total cofactor that was pyruvate–PLP when the proteins were purified from a *ridA* mutant, 66%, 52%, and 98%, from Alr^R209D^, Alr^R209A^, and Alr^R209E^ variants, respectively.

When purified from wildtype cells, the amount of PLP released by the variants differed, suggesting these substitutions also affected the affinity of the protein for the coenzyme. In three of the four variants (Alr^R209S^, Alr^R209D^, Alr^R209A^), less PLP was released in the presence of 2AA stress, *i.e.*, in the *ridA* mutant. The cause of the 2AA impact on PLP levels and the relationship it had to the pyruvate–PLP or the activity of the enzyme was not pursued further here. While each of the variants released significant pyruvate–PLP when purified from the *ridA* mutant, the behavior of Alr^R209E^ was unique. First, the purified Alr^R209E^ variant had very low PLP occupancy. Second, when purified from the *ridA* mutant background, the Alr^R209E^ variant released dramatically more pyruvate–PLP than any of the other proteins, approaching 1 nmol/nmol protein. Third, and most noteworthy, this variant released pyruvate–PLP when purified from the wildtype strain, a condition where persistence of 2AA was not anticipated. In fact, pyruvate–PLP was the dominant form of cofactor released from the Alr^R209E^ variant regardless of whether the protein was purified from the wildtype or *ridA* background (Fig. 3*D* and Table S1).

The release of pyruvate–PLP from Alr^R209E^ purified from the wildtype strain was the first indication that 2AA stress could be present *in vivo* in the presence of active RidA. Further, the data indicated that the Alr^R209E^ variant was more sensitive to, or has a higher affinity for, 2AA than Alr^WT^ or any of the tested variants. Significantly, the R209E substitution results in a protein with the same residue coordinating the pyridine nitrogen as IlvE (transaminase B, EC 2.6.1.42), which has been characterized as an *in vivo* target of 2AA in *S. enterica* (14). In total, the aforementioned results showed the residue coordinating the pyridine nitrogen of PLP impacts the sensitivity of Alr to 2AA. Somewhat surprisingly, all changes made the protein more sensitive to 2AA attack.

### Target protein IlvE confirms 2AA stress in wildtype cells

**Figure 4:**
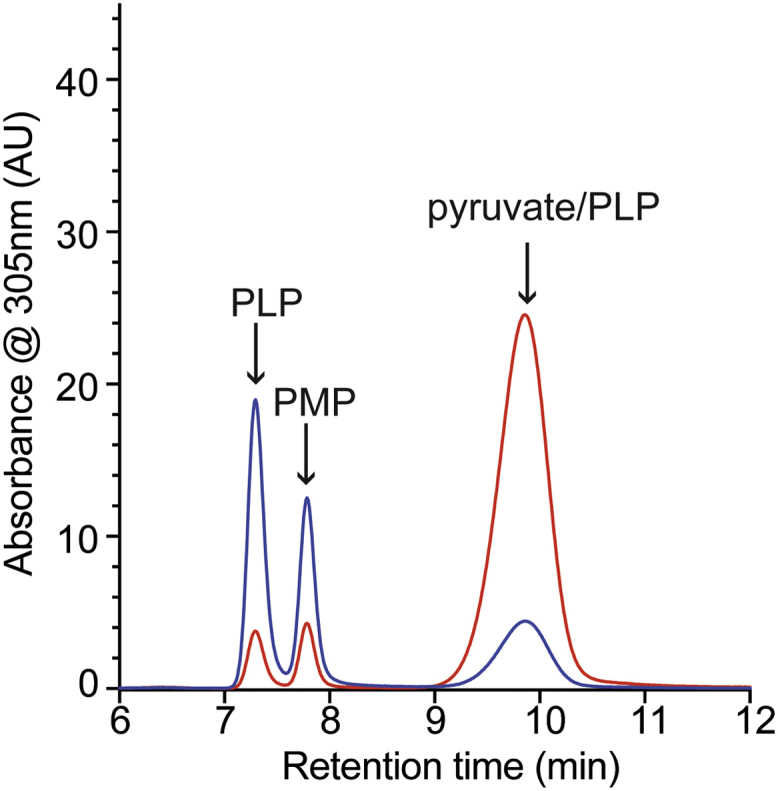
**IlvE is attacked by 2AA with and without the presence of RidA.** IlvE was purified from wildtype (*blue*) and a *ridA* mutant (*red*) strain of *S. enterica*. Cofactors from 0.4 mg of enzyme were released with base treatment and separated by HPLC. Cofactors are labeled based on retention time and coinjection experiments with authentic standards. 2AA, 2-aminoacrylate.

Transaminase B (IlvE) is a target of 2AA stress in *S. enterica* and has been used as a proxy for 2AA-dependent damage in multiple organisms (15, 19, 21, 33). IlvE was purified from the *ridA* and wildtype strains, and the cofactors released by base treatment were analyzed by HPLC (Fig. 4). The IlvE protein purified from the *ridA* mutant released significant pyruvate–PLP, as expected based on the sensitivity of this protein to 2AA in other assays (14, 33). In this sample, pyruvate–PLP was 82% of the total cofactor released. Like Alr^R209E^, IlvE released significant pyruvate–PLP when purified from the wildtype strain, in this case making up 17% the total released cofactor. The pattern of pyruvate–PLP release paralleled that of the Alr^R209E^ variant, with the amount of cofactor released under each condition being similar. Thus, IlvE was the second protein that showed 2AA stress could persist in a wildtype strain. These data suggested that IlvE was more sensitive to 2AA than Alr such that it was damaged by the presumably low level of 2AA that persists in the presence of a functional RidA. This sensitivity justified the continued use of IlvE as to query the 2AA stress level in a cell and indicated that extraction of cofactors provides a more sensitive assay for *in vivo* damage than enzymatic activity.

Interestingly, the cofactor profile released from IlvE showed the presence of two peaks with a retention time near that of PLP, rather than the single peak observed for the other PLP-dependent proteins tested. IlvE is a transaminase, suggesting there would be enzyme subpopulations with either PLP or pyridoxamine 5'-phosphate (PMP) occupancy (34, 35). Coinjection with authentic standards determined that the early peak was PLP, while the one with the later elution time was PMP (Fig. S2). This profile is consistent with a pool of the enzyme available to catalyze each half of the transamination (36).

### Primary 2AA producer, IlvA, can be targeted by 2AA *in vivo*

**Figure 5:**
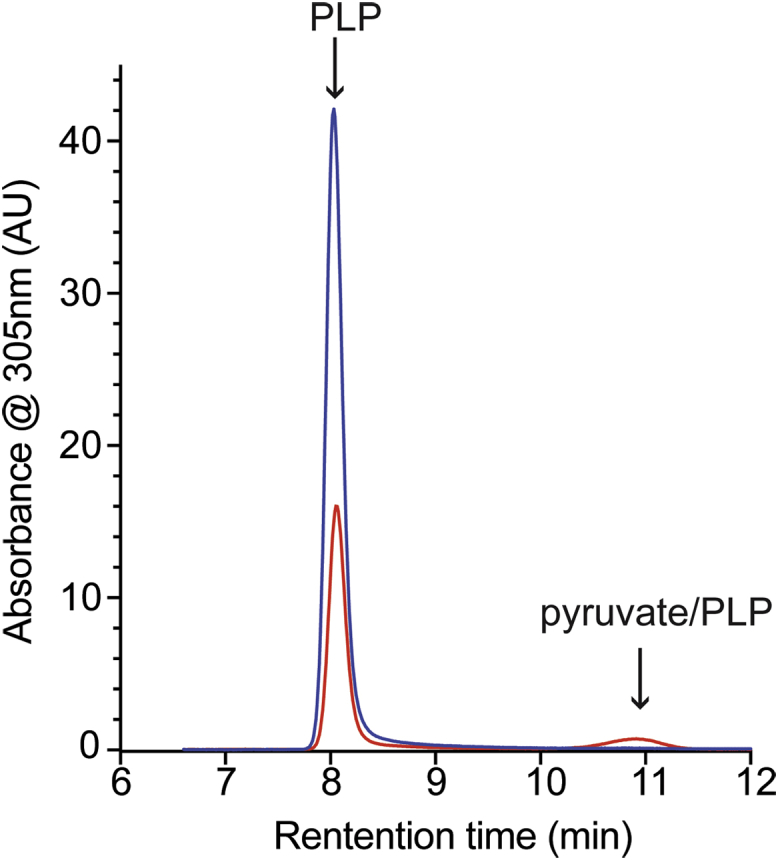
**IlvA is impacted by the 2AA stress it generates.** IlvA was purified from wildtype (*blue*) and a *ridA* mutant (*red*) strain of *S. enterica*. Cofactors from 0.4 mg of enzyme were released with base treatment and separated by HPLC. Cofactors are labeled based on retention time and coinjection experiments with authentic standards. 2AA, 2-aminoacrylate.

**Figure 6:**
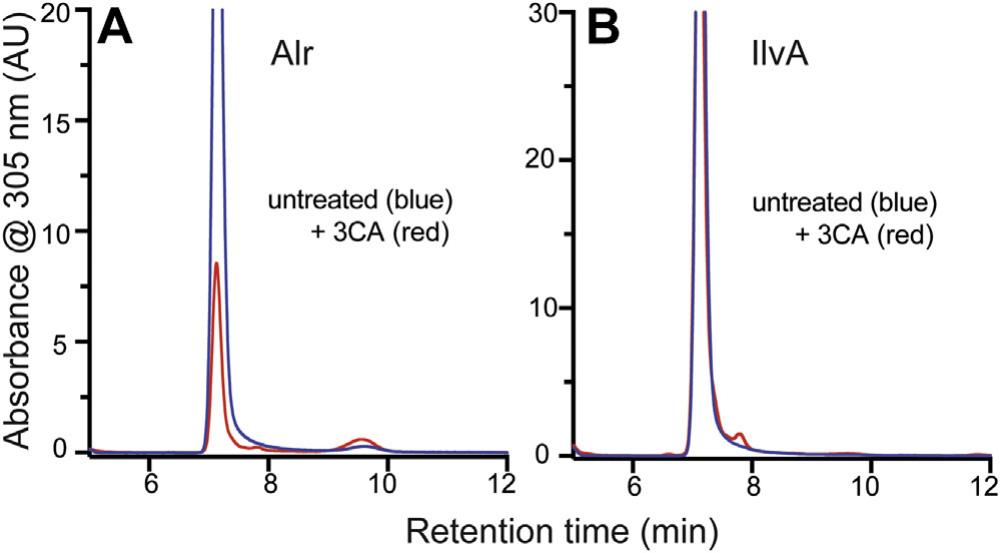
**Alr but not IlvA is sensitive to 3-chloroalanine (3CA).***A* and *B*, proteins Alr (*A*) Alr and IlvA (*B*) were purified from a wildtype (*ridA*+). Proteins were incubated with (*red*) or without (*blue*) 3CA, as described in the text. Cofactors were released with base treatment and separated on HPLC.

*In vivo* and *in vitro* work has shown that serine/threonine dehydratase (IlvA) generates the 2AA that causes phenotypic defects in at least *S. enterica*, *Saccharomyces cerevisiae*, and *Pseudomonas aeruginosa* (reviewed in the study by Irons *et al.* (3)). The generation of 2AA in the catalytic mechanism of serine dehydration suggests that IlvA must be resistant to attack by this reactive enamine. The implication is that either the 2AA generated by IlvA leaves the enzyme active site before it can attack the resident PLP or the active site environment is not conducive to an attack. IlvA was overexpressed and purified from *ridA* mutant and wildtype strains. The IlvA protein purified from wildtype released only the PLP cofactor as expected. However, the protein purified from the *ridA* strain released a small, but detectable, amount of pyruvate-PLP, amounting to 7% of the released cofactor (Fig. 5). It was formally possible that the 2AA IlvA generated can attack PLP at some frequency before it leaves the active site. However, if this scenario were true, the status of RidA should have no impact on the frequency of this occurrence since it does not impact turnover. Thus, under such a scenario, a similar cofactor profile would be expected from both strain backgrounds. Instead, the data support the scenario in which 2AA leaves the IlvA active site. In this situation, when 2AA accumulates in a *ridA* strain, the enamine could re-enter the active site at a low frequency and attack the bound PLP. This scenario would put IlvA in the same situation as the other proteins and supports the idea that IlvA has high resistance to 2AA damage. The interaction of IlvA with the suicide inhibitor 3-chloroalanine (3CA) supported this interpretation. 3CA is known to damage PLP-dependent enzymes when an enzyme initiates the catalysis of 3CA and generates 2AA within the active site and this 2AA inhibits the enzyme (37). Unlike Alr and other 2AA-sensitive proteins (14, 26), incubation of IlvA with 3CA failed to produce formation of pyruvate–PLP, indicating an inability of IlvA to be damaged by endogenously generated 2AA (Fig. 6). While it is formally possible that the release of pyruvate–PLP from IlvA was dependent on overexpression of IlvA, detection of this adduct only when the protein was expressed in a *ridA* mutant reduced this possibility.

## Discussion

Metabolite stress caused by the endogenously generated reactive enamine 2AA was uncovered in *S. enterica* with the characterization of RidA and its role as a 2AA deaminase. Prior to the study here, detection of 2AA stress was based on the phenotypic impact caused by damage to one or more PLP-dependent enzymes and/or decreased specific activity of the damaged protein(s). Past *in vitro* work defined a mechanism by which 2AA could attack PLP in the active site and generate a pyruvate–PLP adduct that could be released and quantified. The experiments described herein took advantage of this property of 2AA damage to query enzymes for their sensitivity to damage by 2AA *in vivo*. The data suggested active site qualities that influence the susceptibility to 2AA attack.

Specifically, our results showed that single substitutions at the R209 residue of Alr, which coordinates the pyridine nitrogen of PLP, dramatically changed the sensitivity to 2AA attack. Interestingly, the ratio of enzyme damaged by 2AA roughly correlated with the electrophilic strength of the Schiff base, using the pyridine N–coordinating residue. The Schiff base, when the PLP pyridine nitrogen is coordinated by the native arginine (R209), is expected to be less electrophilic than each of the substitutions tested. Consistently, among Alr variants tested, the wildtype had the lowest level of 2AA damage as measured by the ratio of pyruvate–PLP to PLP released from the proteins exposed to *in vivo* 2AA stress (Table 1). In other words, 2AA damage to Alr variants correlated well with the electrophilicity of the Schiff base N. This finding is supported by previous observations using variants of tryptophan synthase, where substitutions at the pyridine N–coordinating residue expected to increase the electrophilicity of the Schiff base N (S377D and S377E) led to increased inactivation by 2AA produced from L-serine or 3-chloro-L-alanine substrate, as compared to wildtype enzyme (38). Future analyses of more Alr variants and variants of other enzymes are necessary to extend this correlation and better understand parameters of an active site that contribute to the sensitivity to 2AA attack. Specifically, the sensitivity of variants that change the residue coordinating the 3′O would be informative, as this residue is also predicted to influence Schiff base N electrophilicity (32).

**Table 1:** Cofactor content of different protein samples

Relevant host genotype	Protein	PLP	PMP	Pyruvate–PLP	% Adduct
WT	Alr	0.87 ± <0.01	ND	ND	N/A
*ridA*	Alr	0.70 ± <0.01	ND	0.11 ± <0.01	14%
WT	Alr^R209S^	0.46 ± <0.01	ND	ND	N/A
r*idA*	Alr^R209S^	0.29 ± <0.01	ND	0.54 ± <0.01	65%
WT	Alr^R209E^	0.05 ± <0.01	ND	0.19 ± <0.01	79%
*ridA*	Alr^R209E^	0.02 ± <0.01	ND	0.98 ± <0.01	98%
WT	Alr^R209D^	0.18 ± <0.01	ND	ND	N/A
*ridA*	Alr^R209D^	0.18 ± <0.01	ND	0.33 ± <0.01	65%
WT	Alr^R209A^	0.78 ± <0.01	ND	ND	N/A
*ridA*	Alr^R209A^	0.30 ± <0.01	ND	0.30 ± <0.01	50%
WT	IlvA	1.25 ± 0.04	ND	ND	N/A
*ridA*	IlvA	0.45 ± 0.02	ND	0.03 ± <0.01	6%
WT	IlvE	0.30 ± <0.01	0.33 ± <0.01	0.12 ± <0.01	27%
*ridA*	IlvE	0.07 ± <0.01	0.11 ± <0.01	0.72 ± <0.01	80%

The relevant proteins were expressed and purified from the indicated host strain. Cofactors were released from each sample with base treatment and separated on HPLC. The amount of each cofactor (nmol/nmol protein) is reported. The % adduct column reports the ratio of pyruvate–PLP to pyruvate–PLP + PLP. Proteins were expressed in cells grown in minimal glucose containing 1 mM glycine, 50 μM pyridoxine, and 15 μg/ml ampicillin with supplementation of 0.2% (wt/vol) L-arabinose to induce expression of the protein-encoding gene and 5 mM L-serine to induce 2AA production, as described in the text.

Abbreviations: N/A, not applicable; ND, not detected.

An unexpected result from experiments described herein showed that 2AA stress existed in the presence of functional RidA, a condition previously thought to prevent persistence of 2AA. Both a variant of Alr and wildtype IlvE showed scars of 2AA damage, even in the presence of a functional RidA. Further analyses are required to assess the source and level of 2AA that is present in the wildtype strain, compared to a *ridA* mutant. The system defined here will facilitate these studies and provides sensitivity that is not allowed with phenotypic or activity assay approaches. Our current hypothesis is that RidA is unable to completely clear the cell of 2AA stress. These data suggested that by analyzing the scars of 2AA damage on proteins in a wildtype strain, the proteins that are most sensitive to attack by 2AA could be identified.

In total, this study refined a model protocol and provided proof of concept that it can be used to query proteins from diverse systems for their sensitivity to 2AA *in vivo*. Thus far, in addition to *S. enterica* proteins, PLP-dependent proteins from yeast (Hem1p (26)) and *P. aeruginosa* (IscS (R. L. Fulton, unpublished results)) were found to be sensitive to 2AA damage, when expressed in the *S. enterica* system. Importantly, these results were predicted by phenotypic analysis of the relevant loci in their native organism. In addition, this system has the potential to further studies to manipulate conditions and query the level of 2AA persistence *in vivo*, analyze hierarchy of enzyme sensitivity, and assess the potential of different proteins, both RidA and others, to eliminate 2AA stress. In total, the data herein highlight the potential for this system to generate insights into 2AA stress and how it impacts specific proteins to affect cellular fitness in diverse organisms.

## Experimental procedures

### Bacterial strains and media

Strains used in this work are derivatives of *S. enterica* subsp. *enterica* serovar Typhimurium strain SB300AI (39) or LT2 and are described in Table S1. DM13509 was generated by transducing *S. enterica* SB300AI to His^+^. Other markers were moved by standard genetic techniques to generate strains of the appropriate genotype. Minimal medium was no-carbon E (NCE) supplemented with 1 mM MgSO_4_ (40), trace minerals (41), and 11 mM D-glucose as the sole carbon source. Difco nutrient broth (8 g/l) containing NaCL (5 g/l) and superbroth (SB; 32 g tryptone, 20 g yeast extract, 5 g sodium chloride, and 5 ml 1 N sodium hydroxide/l) were used as rich medium. Difco BiTek agar (15 g/l) was added for solid medium. Antibiotics were used at the following concentrations for rich (or minimal) medium: kanamycin, 50 (12.5), tetracycline, 20 (10), and ampicillin 150 (15) μg/ml. All chemicals were obtained from Sigma.

### Site-directed mutagenesis

Derivatives of pET20b (Novogen) expressing Alr (pDM1602) or IlvA (pDM1578 (42)) with an N-terminal His6 tag were constructed by standard methods. Site-directed mutations in the relevant gene were generated according to the single primer polymerase chain reaction “QuikChange” method (43). Briefly, for a 50-μl reaction, 1 ng/μl of template plasmid DNA was incubated with 0.01 nmol of the appropriate primer (Table 1) and amplified for 30 cycles using the Q5 high-fidelity DNA polymerase (New England Biolabs). Fragments were treated with *Dnp*1 (New England Biolabs) and allowed to incubate at 37 °C for 2 h before transformation into *E. coli* DH5α cells where successful transformants were selected on rich medium containing ampicillin. All mutations were sequence verified by Eton Biosciences.

### Protein purification

Proteins were purified from *S. enterica* strains DM13509 and DM17050, which encode T7-polymerase under the control of the araBAD promoter (39). The relevant proteins were encoded in pET20b derivative plasmids, except IlvE, which was in plasmid pET28b. For each purification, two 10-ml cultures were grown in SB medium with ampicillin to full density at 37 °C. Each culture was used to inoculate 1.5 l of minimal glucose medium containing glycine (1 mM) to enhance growth (17), pyridoxine (50 μM) to ensure PLP excess, and ampicillin in a 2.8-l baffled Fernbach flask and incubated at 30 °C with shaking (180 RPM). When the cultures reached an *A*_650_ of ∼0.6, the following were added: L-serine (5 mM) to induce 2AA production (14), additional glycine (1 mM) to increase growth, and L-arabinose (0.2% wt/vol) to induce expression of gene encoding the relevant protein. Incubation continued at 25 °C until full density (*A*_650_ of ∼1.2) was reached (∼15 h). Cells were pelleted by centrifugation (10,000*g*, 4 °C, 10 min) and stored at −80 °C for future use. Prior to purification, cells were resuspended (2 ml/1 g) in buffer (50 mM KPO_4_, pH 8, 100 mM NaCl, 20 mM imidazole, and 10% glycerol). The cell suspension was incubated with lysozyme (1 mg/ml) and DNase (125 mg/ml) for 30 min on ice prior to disruption by a One Shot Cell Disrupter at 18,000 psi. The resulting lysate was centrifuged (48,000*g*, 45 min at 4 °C), and the supernatant passed through a 0.45-μM polyvinylidene difluoride filter. The clarified lysate was loaded on a 5-ml HisTrap HP column and purified according to the manufacturer’s protocol (GE Healthcare). Briefly, the protein was bound and washed with buffer/binding buffer (100 mM KPO_4_, pH 8, 100 mM NaCl, 20 mM imidazole, 10% glycerol) and eluted with a linear gradient from 100% binding buffer to 100% elution buffer (100 mM KPO_4_, pH 8, 100 mM NaCl, 500 mM imidazole, and 10% glycerol) over ten column volumes. Three-milliliter fractions were collected over 25 min, and those with significant protein (a.k.a. absorbance at 280 nm) after the washing step were pooled and concentrated using a 30,000-Da Ultra-15 centrifugal filter (Amicon). The protein was desalted into storage buffer (100 mM KPO_4_, pH 8.0, 100 mM NaCl, 10 μM PLP, and 10% glycerol) using a PD-10 desalting column (GE Healthcare). The purified protein was frozen by dripping into liquid nitrogen and stored at −80 °C until use. A typical purification yielded proteins of ∼95% purity. Given the high purity of the purified proteins, concentration was determined using the extinction coefficient determined with https://web.expasy.org/protparam/ (44).

### Pyruvate–PLP adduct synthesis

Authentic Pyruvate–PLP adduct was generated as described (16, 45). Briefly, PLP (1.5 mM) and pyruvate (6 mM) reacted in 0.5 M KOH (total volume of 80 ml) overnight at room temperature with constant stirring. The solution was neutralized with 70% perchloric acid (∼3–4 ml), filtered with a 0.45-μM PDVF membrane, and the resulting filtrate lyophilized. The resulting residue, which contained PLP and pyruvate–PLP adduct, was resuspended in 2 ml of water. Pyruvate–PLP and PLP were separated by HPLC. A 10-μl sample was passed over a Luna C_18_ (250 by 4.6 mm) column (Phenomenex) at 0.8 ml/min with the 2-step isocratic method: 0 to 5 min 100% buffer A (0.06% trifluoroacetic acid [TFA]), 5 to 17 min 97% buffer A and 3% buffer B (100% methanol). Eluent was monitored at 283 nm, the maximum absorbance for the adduct.

### Cofactor characterization with HPLC

Reactions containing 0.4 mg of enzyme and 30 mM KOH in a total volume of 100 μl were allowed to incubate for 10 min at room temperature. The protein was denatured by the addition of 10% TFA until a visible precipitate was formed. The solution was centrifuged for 3 min (16,000*g*), and the resulting supernatant was filtered through a 0.45-μM membrane filter. Visualization of the cofactor content was performed using a HPLC 2-step isocratic method with a Luna C_18_ (250 by 4.6 mm) column (Phenomenex) (16): 0 to 5 min 0.8 ml/min 100% buffer A (0.06% TFA), 5 to 17 min 0.8 ml/min 97% buffer A and 3% buffer B (100% Methanol). Eluent was monitored at 305 nm using Shimadzu Lab Solutions software since that minimizes the background while showcasing clear and separated peaks of PLP and pyruvate–PLP adduct (16). Based on the retention time, coinjection of authentic compounds, UV-visible spectra, and verification in a previous study, the peak with the retention time of around 9.5 to 11.5 min was assigned as pyruvate–PLP (16). In the case of cofactor extraction from IlvE proteins, the coinjection of authentic compounds as well as the UV-visible spectra determined that the first peak eluted is PLP while the second is PMP (Fig. S3).

### Alr activity assay

Racemization of D-alanine to L-alanine by the purified protein was assayed by modifying a previously described protocol (46). Briefly, the assay mixture contained 50 mM glycine (pH 9), 1.2 mM NAD^+^, 20 mM D-alanine, 0.1 unit *Bacillus subtilis* L-alanine dehydrogenase (Sigma-Aldrich), and approximately 0.4 μg of Alr in a total volume of 105 μl. Reduction of NAD+ was monitored by the increase in absorbance at 340 nm. The extinction coefficient for NADH in 50 mM glycine (pH 9.0) was determined in the previous publication (16) (ε = 4000 absorbance units [AU] M^−1^ cm^−1^) and used to calculate the rate of L-alanine production. Reactions were performed at room temperature, and the specific activities are reported as μmol alanine per min per mg.

## Data availability

The data that support the findings of this study are within the manuscript or in the supplementary material of this article, and additional information is available upon request from the authors.

## Supporting information

This article contains supporting information (1).

## Conflict of interest

The authors declare that they have no conflicts of interest with the contents of this article.
