# Supplementary material for: 2-Aminoacrylate stress damages diverse PLP-dependent enzymes in vivo
Source: J Biol Chem. 2022 Apr 20;298(6):101970. doi: 10.1016/j.jbc.2022.101970 (PMC9127364; doi:10.1016/j.jbc.2022.101970)
Supplement: Supplemental Figures S1–S3 and Table S1 [file mmc1.pdf]

SUPPORTING INFORMATION

2-Aminoacrylate stress damages diverse PLP-dependent enzymes *in vivo*

Wangchen Shen, Andrew J. Borchert, and Diana M. Downs

Table S1

Figure S1

Figure S2

Figure S3

Literature Cited.

1. McKinney, J., et al., *Tightly regulated gene expression system in Salmonella enterica serovar Typhimurium.* J. Bacteriol., 2002. **184**(21): p. 6056-9.
2. Way, J.C., et al., *New Tn10 derivatives for transposon mutagenesis and for construction of lacZ operon fusions by transposition.* Gene, 1984. **32**(3): p. 369-379.

TABLE S1. Bacterial strains, plasmids, and primers

Strain	Genotype
DM10000	*Salmonella enterica* LT2 (Wildtype)
DM13509[1]	*aadA::araC*-P_{BAD}-T7*pol*$^+$ *xyL rpsL*
DM17050	*aadA::araC*-P_{BAD}-T7*pol*$^+$ *xyL rpsL ridA1*::Tn10(d)[2]
DM17051	*aadA::araC*-P_{BAD}-T7*pol*$^+$ *xyL rpsL ridA1*::Tn10(d) / pDM1602
DM17053	*aadA::araC*-P_{BAD}-T7*pol*$^+$ *xyL rpsL ridA1*::Tn10(d) / pDM1507
DM17181	*aadA::araC*-P_{BAD}-T7*pol*$^+$ *xyL rpsL* pDM1507
DM17182	*aadA::araC*-P_{BAD}-T7*pol*$^+$ *xyL rpsL* pDM1504
DM17183	*aadA::araC*-P_{BAD}-T7*pol*$^+$ *xyL rpsL ridA1*::Tn10(d) / pDM1504
DM17184	*aadA::araC*-P_{BAD}-T7*pol*$^+$ *xyL rpsL* / pDM1505
DM17185	*aadA::araC*-P_{BAD}-T7*pol*$^+$ *xyL rpsL ridA1*::Tn10(d) / pDM1505
DM17186	*aadA::araC*-P_{BAD}-T7*pol*$^+$ *xyL rpsL* /pDM1506
DM17187	*aadA::araC*-P_{BAD}-T7*pol*$^+$ *xyL rpsL ridA1*::Tn10(d) / pDM1506
DM17292	*aadA::araC*-P_{BAD}-T7*pol*$^+$ *xyL rpsL* / pDM1579
DM17293	*aadA::araC*-P_{BAD}-T7*pol*$^+$ *xyL rpsL ridA1*::Tn10(d) / pDM1579
DM17294	*aadA::araC*-P_{BAD}-T7*pol*$^+$ *xyL rpsL* / pDM1577
DM17295	*aadA::araC*-P_{BAD}-T7*pol*$^+$ *xyL rpsL ridA1*::Tn10(d) / pDM1577
DM17296	*aadA::araC*-P_{BAD}-T7*pol*$^+$ *xyL rpsL* / pDM1578
DM17297	*aadA::araC*-P_{BAD}-T7*pol*$^+$ *xyL rpsL ridA1*::Tn10(d) / pDM1578

Plasmid	Description
pDM1578	pET20b-*ilvA*
pDM1602	pET20b-*alr* (wildtype)
pDM1504	pET20b-*alr57* (R209E)
pDM1505	pET20b-*alr56* (R209A)
pDM1506	pET20b-*alr55* (R209D)
pDM1507	pET20b-*alr54* (R209S)
pDM1577	pET28b-*ilvE*

Table S1 (cont)

Primer	Sequence
LT2_*alr*_R209D	5’-agtctcactttgactgggcgGATccgggcatcattttgtatgg-3’
LT2_*alr*_R209A	5’-agtctcactttgactgggcgGCGccgggcatcattttgtatgg-3’
LT2_*alr*_R209E	5’-agtctcactttgactgggcgGAAccgggcatcattttgtatgg-3’
LT2_*alr*_R209S	5’-agtctcactttgactgggcgAGCccgggcatcattttgtatgg-3’

[1]A His+ derivative of the SB300AI *S. enterica* strain harboring the chromosomally borne gene for T7 polymerase under control of the arabinose-inducible P_{BAD} promoter (*aadA::araC*-P_{BAD}-T7*pol*$^{+}$) [1].

[2]Tn10(d) refers to the transposition-defective mini-Tn10 (Tn10Δ16Δ17) [2].

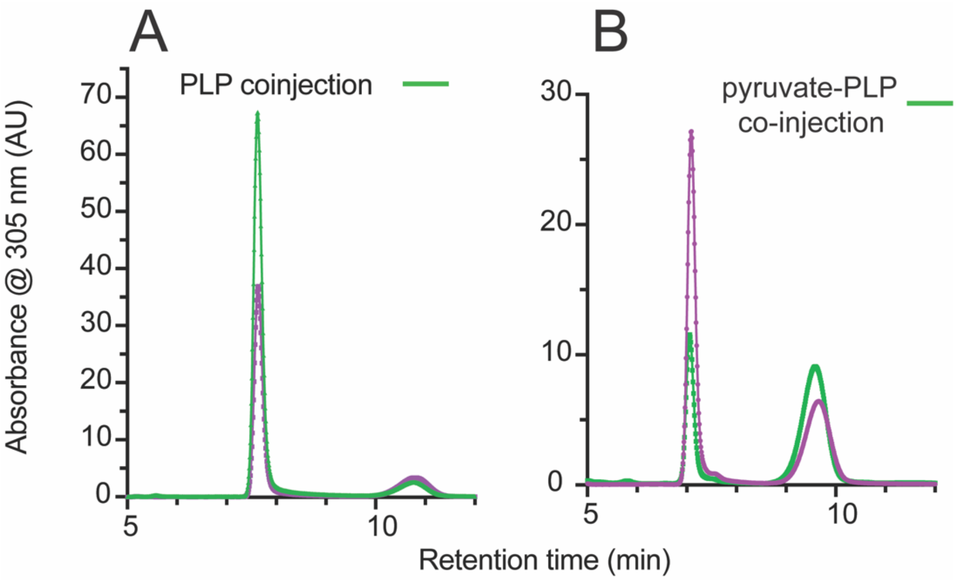

Figure S1. Assignment of peaks to PLP and pyruvate-PLP. Alr was purified from a *ridA* background, the cofactors were released from 0.4 mg of enzyme with base, and separated on HPLC as described (purple). Green chromatogram tracing shows the released cofactors with added PLP(A) or pyruvate-PLP (B). In each case the cofactors released from Alr took up half of the total volume and concentrated cofactor (PLP or PLP/pyruvate adduct) generated the other half of the volume.

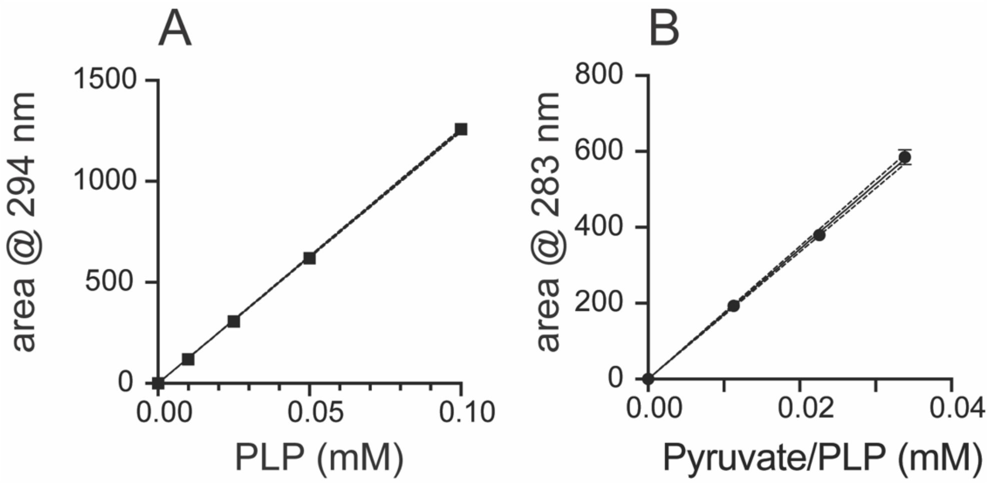

Figure S2. Standard cure for PLP and pyruvate-PLP. The standard curves for (A) PLP and (b) pyruvate/PLP are plotted. The y axis is the area of each peak at a known concentration and the x axis are the corresponding concentrations. The area of PLP and PLP/pyruvate are monitored at their individual absorbance maximum, which for PLP is 294nm, and for PLP/pyruvate, 283nm. (The R square value for both is greater than 0.99).

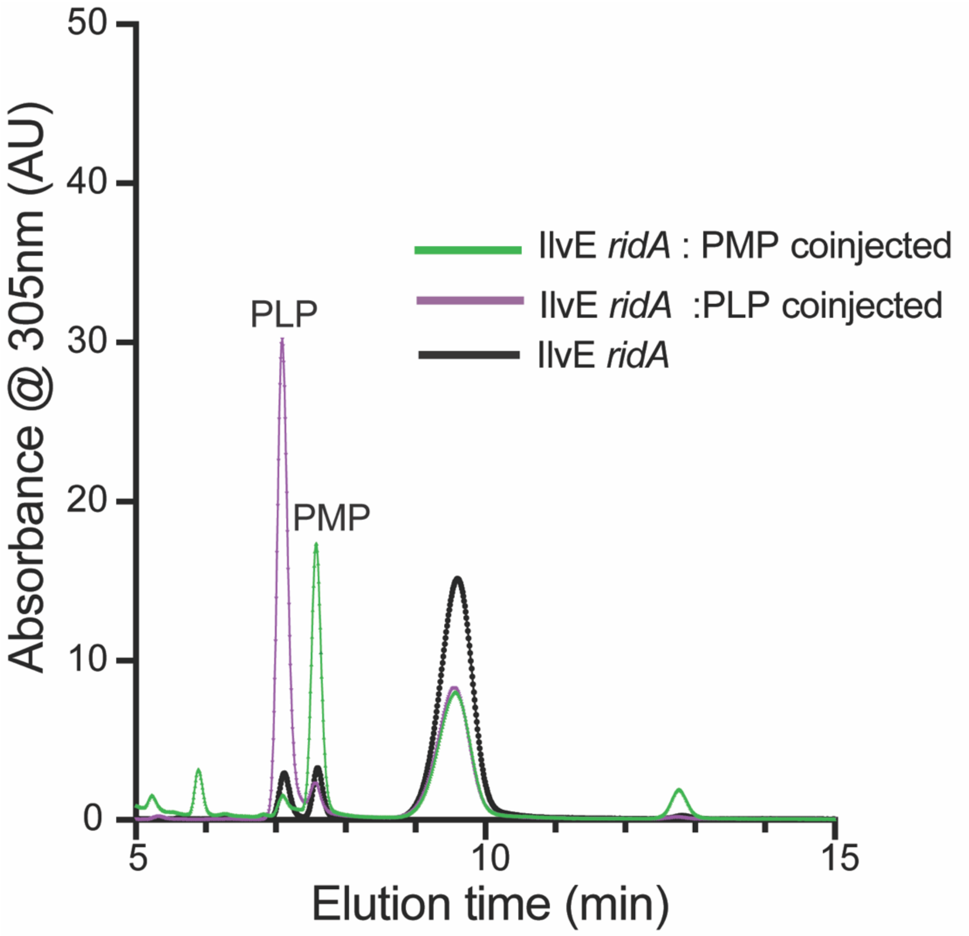

Figure S3. Peak assignment for PMP. The chromatogram of the cofactors released from IlvE purified from a *ridA* strain(black). The same sample including PLP (purple) or PMP (green).
